# Daylight Saving Time and Spontaneous Deliveries: A Case–Control Study in Italy

**DOI:** 10.3390/ijerph17218091

**Published:** 2020-11-03

**Authors:** Rosaria Cappadona, Sara Puzzarini, Vanessa Farinelli, Piergiorgio Iannone, Alfredo De Giorgi, Emanuele Di Simone, Roberto Manfredini, Rosita Verteramo, Pantaleo Greco, María Aurora Rodríguez Borrego, Fabio Fabbian, Pablo Jesús López Soto

**Affiliations:** 1 Department of Medical Sciences, University of Ferrara, 44121 Ferrara, Italy; rosaria.cappadona@unife.it (R.C.); pg.iannone88@gmail.com (P.I.); pantaleo.greco@unife.it (P.G.); 2 Obstetrics & Gynecology Unit, Department of Reproduction and Growth, Azienda Ospedaliero-Universitaria “S. Anna”, 44124 Ferrara, Italy; s.puzzarini@ospfe.it (S.P.); vanessa.farinelli@edu.unife.it (V.F.); r.verteramo@ospfe.it (R.V.); 3 Department of Nursing, Instituto Maimónides de Investigación Biomédica de Córdoba (IMIBIC), 14071 Córdoba, Spain; en1robom@uco.es (M.A.R.B.); n82losop@uco.es (P.J.L.S.); 4 Clinica Medica Unit, Department of Medicine, Azienda Ospedaliero-Universitaria “S. Anna”, 44124 Ferrara, Italy; degiorgialfredo@libero.it; 5 Department of Biomedicine and Prevention, University of Rome Tor Vergata, I-00133 Rome, Italy; emanuele.disimone@uniroma1.it; 6 Department of Nursing Pharmacology and Physiotherapy, University of Córdoba, 14071 Córdoba, Spain

**Keywords:** daylight saving time (DST), desynchronization, circadian rhythm, chronobiology, nursing, spontaneous delivery, midwifery, obstetrics

## Abstract

(1) Background: Although the current literature shows that daylight saving time (DST) may play a role in human health and behavior, this topic has been poorly investigated with reference to Obstetrics. The aim of this case–control study was to evaluate whether DST may influence the number of spontaneous deliveries. (2) Methods: A low-risk pregnancy cohort with spontaneous onset of labor (*n* = 7415) was analyzed from a single Italian region for the period 2016–2018. Primary outcome was the number of spontaneous deliveries. Secondary outcomes were: gestational age at delivery, type and time of delivery, use of analgesia, birth weight, and 5-min Apgar at delivery. We compared the outcomes in the two weeks after DST (cases) to the two weeks before DST (controls). (3) Results: Data showed no significant difference between the number of deliveries occurring before and after DST (Chi-square = 0.546, *p* = 0.46). Vaginal deliveries at any gestational age showed no statistical difference between the two groups (Chi-square = 0.120, *p* = 0.73). There were no significant differences in the secondary outcomes, as well. (4) Conclusions: DST has neither a significant impact on the number of deliveries nor on the obstetric variables investigated by this study.

## 1. Introduction

The mechanism that starts the spontaneous onset of labor still remains an unanswered question in the current literature [1]. In the obstetric field, scientific research is rich in studies, conducted both in vitro and in vivo, explaining the action of hormones in the onset and maintenance of labor during birth by correlating it with the circadian cycle [2]. Cortisol is the main stress hormone responsible for the normal adaptation of the neonate to extrauterine life. Placental corticotrophin releasing hormone (CRH) is active from the early weeks of pregnancy and determines its duration, the time of onset of labor, and the timing of delivery; moreover, its plasma levels increase rapidly near the beginning of spontaneous labor. Data suggest that CRH may have a pivotal role in activating the mechanism of delivery [3,4,5]. In addition, type of delivery, e.g., vaginal or caesarean, may determine a different stress response. Recently, a study compared the activity of 11beta-hydroxysteroid dehydrogenase type 2 (11β-HSD 2) in the placenta and the umbilical cord blood cortisol level between caesarean sections, with or without uterine contraction, and vaginal delivery groups [6]. No statistically significant differences in the activity of 11β-HSD 2 were found in placentas delivered via caesarean sections compared to vaginal deliveries, while umbilical cord blood cortisol in the elective caesarean sections group was significantly lower compared to the vaginal deliveries and intrapartum caesarean sections [6]. Sleep disturbances and alterations in the circadian rhythm can lead to an increase in the levels of stress hormones and the activation of a pro-inflammatory condition can trigger and enhance the activity of prostaglandins and oxytocin, by promoting uterine contractions, the dynamic phenomena of labor, and consequently, the onset of labor [4,5,6,7]. These alternations can also be induced by shift work; in fact, women who experience an alteration of their circadian rhythm due to work are significantly more exposed to a reduction in birth rate and a lower birth weight of their newborns [8]. The purpose of the so-called “Summer time” was to capitalize on natural daylight: by turning the clock one hour forward as the days get longer in Spring, sunset is delayed by this same hour, until the clock is set back again in Autumn. After a first phase of Daylight Saving Time (DST) policy, where single countries decided their own standards, not regulated, harmonization attempts began in the 1970s to facilitate the effective operation of the internal market, and this practice is applied in over 60 countries worldwide [9]. Since light–dark alternation influences the synchronization of circadian rhythms of most human systems, DST may cause a chrono-disruption by significantly altering circadian rhythm and negatively influencing sleep quality [10]. A growing amount of evidence showed that DST transition may have negative effects on health [11,12,13,14,15,16]. Thus, based on these premises, an international consensus statement suggested that DST cannot be encouraged and therefore, should be discontinued [17].

As for female reproduction, disruptions of circadian rhythms, e.g., shift work, jet lag, and DST, have been associated with poorer fertility and early pregnancy outcomes [18]. However, very few data are available on the effect of DST on the onset of spontaneous labor. Only one recent published study showed no significant association between DST circadian rhythm and the number of spontaneous deliveries [19]. Based on this hypothesis, we evaluated whether DST might affect the number of spontaneous deliveries in a single Italian region.

## 2. Materials and Methods

**Figure 1:**
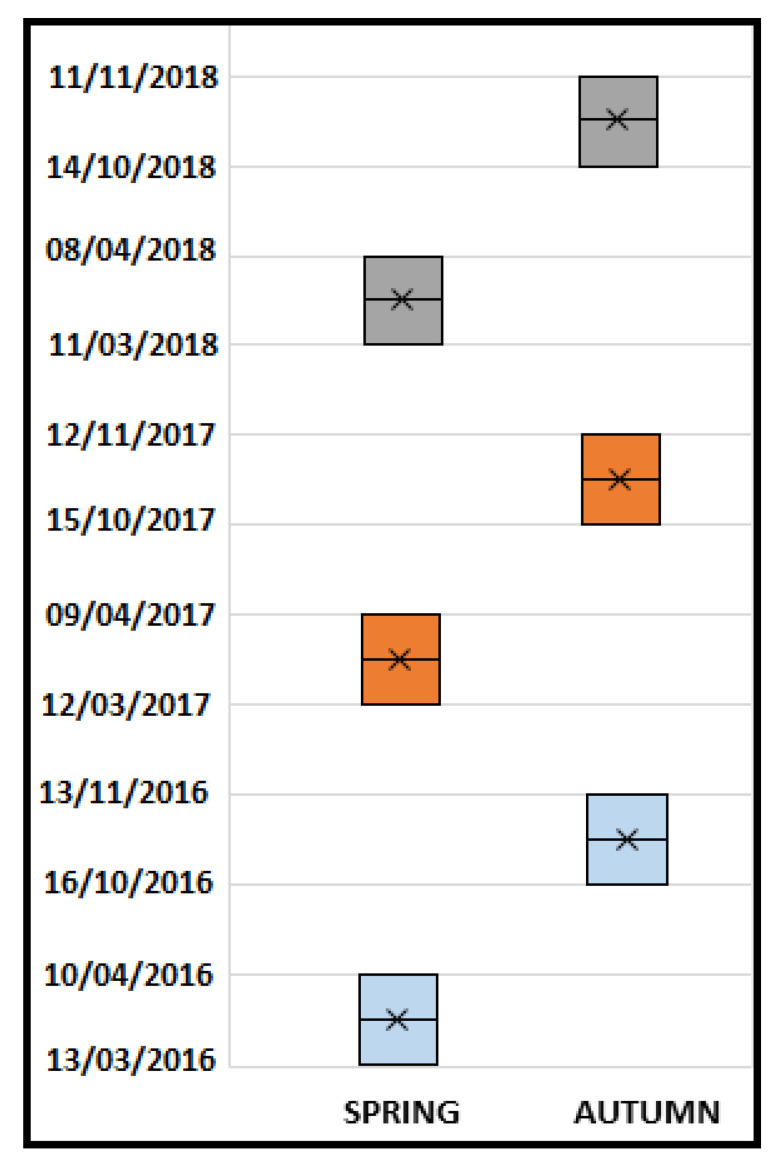
The two weeks belonging to the control period and the exposure period identified by daylight saving time (DST) during each year analyzed (2016–2018) are reported (grey is related with 2016, orange with 2017 and light blue with 2018).

A cohort of 7415 deliveries from low risk pregnancies with spontaneous onset of labor was enrolled in this case–control study. We used the Certificate of Childbirth Assistance (CedAP) data collection from one single Italian region, Emilia-Romagna, in the period 2016–2018. We identified the exact dates of DST during each year analyzed (Figure 1). Age and gestational age were evaluated. We defined the 2 weeks after DST as the exposure period and the 2 weeks before DST as the control period.

Exclusion criteria are shown in Table 1.

The primary outcome was defined as the number of deliveries, in particular the number of non-operative vaginal deliveries.

The rationale underlying the choice of the latter outcome was to exclude, as far as possible, the conditioning acted out by the health workers and the healthcare organizational system that weighs on the birth path, in order to bring out the influences that the endocrine and central nervous systems have on childbirth.

Secondary outcomes were:

- Gestational age delivery, defining preterm before 37 weeks, at term between 37 and 41 weeks, and post term beyond 41 weeks;
- Type of delivery: vaginal delivery, cesarean section, or operative delivery;
- Time of delivery: time when the birth occurred, combining three moments of the day—morning (08:00–15:59), afternoon (16:00–23:59), or night (00:00–07:59);
- Birth weight, divided into three distinct classes: less than 2500 g, between 2500 and 4000 g, and more than 4000 g;
- Five-minute Apgar at birth;
- Use of analgesia in labor.

We also analyzed local data relating to individual provinces. In particular, the number of at term deliveries that occurred in the three-year period was considered in the various Emilia-Romagna provinces, in order to verify whether there were any differences related to DST. The statistical analysis of the data was performed with the “chi-square” test.

We considered a 5% error with a confidence interval of 95%, standard deviation of 0.5, and z-score of 1.96.

The differences in the number of deliveries across the Spring and Autumn shifts were assessed for significance using the Poisson Means Test.

Annual birth rate was calculated as the ratio between the number of births during the two weeks pre and post DST, and the mean value of births obtained by the sum of births in the previous and the considered year divided by 2. The ratio was then multiplied by 10^3^. This calculation was performed for any year considered in this study.

(1) $$N\;\left(x\right)=\frac{N\left(x\right)}{\left[\begin{array}{c} \frac{B\left(x-1\right)}{2}+\frac{B\left(x\right)}{2} \end{array}\right]}*1000$$

N = number of births during the index 2 weeks;

X = year considered;

x−1 = previous year;

B = total births in the Emilia-Romagna region of Italy.

In addition, we calculated the age and gestational age of participants. A logistic regression analysis was carried out, where the period including the two weeks pre and post DST was the dependent variable and the other parameters investigated in this study the independent ones. A 2-sided *p*-value < 0.05 was considered statistically significant. IBM Statistical Package for Social Science (SPSS 13.0 for Windows, SPSS Inc., Chicago, IL, USA) was used.

## 3. Results

The mean age of the 7415 women was 31.4 ± 5 years, and mean gestational age was 39.3 ± 1.4 weeks. Italian patients totaled 4801 (64.7%), whilst 35.3% were classified as non-Italian. The mean age of the women who delivered during the two weeks before DST were older than those who delivered during the two weeks post DST (31.6 ± 5.4 vs. 31.3 ± 5.3 years, *p* = 0.033); on the contrary, gestational age was not different in the two groups (39.3 ± 1.4 vs. 39.3 ± 1.4 weeks, *p* = ns). As regarding time of delivery, 2708 births occurred in the morning (36.5%), 2022 (27.3%) in the afternoon, and 2685 (36.2%) at night. Birth rate during the Spring and Autumn shifts in the three different years of the study are shown in Table 2.

### 3.1. Primary Outcome

Our results showed a difference in the number of deliveries between the exposed and the control groups, although it did not reach statistical significance (Table 3).

No statistical difference has been observed in the analysis of the number of deliveries (*p* = 0.46), including mode of delivery (vaginal delivery, cesarean section, or operative delivery) and gestational age (term, preterm, post term). The same result was obtained analyzing the number of spontaneous deliveries (*p* = 0.73).

The differences in the number of deliveries and number of spontaneous deliveries across the Spring and Autumn shifts assessed by the Poisson Means Test were not significant (*p* = 0.37, *p* = 0.92, *p* = 0.6, and *p* = 0.65 respectively).

### 3.2. Secondary Outcome

There were no significant differences in the secondary outcomes analyzed, between what was detected during the exposure period and what was found in the control period (Table 4).

Women’s age was independently associated with delivery during the two weeks post DST (OR 1.010, 95% Confidence intervals 1.002–1.019, *p* = 0.021). All the other investigated parameters were not associated with the period of the DST. Therefore, every year of increasing age of the delivering woman increased the risk of event occurrence during the two weeks after DST of 1.0% (Table 5).

Finally, we assessed the percentages of type and time of delivery during the Spring and Autumn shifts, but we could not find any difference (data not shown).

## 4. Discussion

The present study found no statistically significant differences in the number of deliveries that occurred during the two weeks following DST compared to those occurring prior to the DST shifts. The same result was obtained for each secondary outcome analyzed. These data confirm the findings by Laszlo et al. [19] also for the latitudes and climatic conditions of the Emilia-Romagna region of Italy, despite the difference in daily average hours of sunlight (approximately 180 and 50 monthly sun-hours in March and October, respectively, in Sweden, vs. 190 and 100 in March and October, respectively, in Italy). The Swedish study, in fact, showed that the number of daily and weekly deliveries in the case and control group was similar, and statistically significant (IR 1.005, 95% CI 0.990–1.019), underlining the validity of the result only for latitudes, exposure to light, and climatic conditions to which the people included in the sample are subjected. Although the characteristics just stated for Sweden and the Emilia-Romagna region are extremely different, in both studies, it is confirmed that the transition from standard time to summer time and vice versa does not impact on the number of deliveries.

These results confirm data from Roizen et al. [20] regarding the role of oxytocin in the onset and completion of childbirth and its rhythmicity. Hormone secretion, in fact, has its own circadian rhythm, not influenced by the alternation of dark and light and, therefore, not affected by the change of time. This may explain why the number of deliveries in the two weeks following the transition from standard time to summer time, and vice versa, is almost superimposable to that found in the two weeks preceding it.

The analysis of type of delivery was based on the hypothesis of a possible influence of DST on the secretion of melatonin [21,22,23,24], through the indirect evaluation of the outcome of contractile activity. In this case, we could expect to observe a greater number of operative deliveries and cesarean sections in the weeks following DST shifts, caused by dynamic or mechanical dystocia induced by anomaly or stop of contractions. This difference was more marked in the Spring period as the transition from winter time to summer time leads to the loss of one hour of darkness, with a consequent increase in light exposure and reduction in serum melatonin levels and, presumably, also in valid contractions. However, our data do not allow confirmation of this hypothesis; both the statistical analysis performed on the number of cesarean sections (*p* = 0.08) and that relating to the operative deliveries (*p* = 0.99) do not allow us to exclude that what has been detected is not attributable to simple chance.

Another relevant finding in the secondary outcome analysis was the confirmation of previous data collected in Texas, USA, by Mancuso et al. [25]. In our sample also, relating to the Emilia-Romagna region, the transition from standard time to summer time does not significantly affect the time of delivery, regardless of the gestational period and the way it occurs.

Neonatal birth weight was also investigated as a secondary outcome, to test the hypothesis of a short-term, acute effect of rhythm disruption. This is because the available data in the literature refer to studies performed on shift worker women [8], and shift work represents a chronic, long-term condition of rhythm desynchronizing effect. According to this hypothesis, the expected result could be, therefore, an increase in newborns with a low birth weight (<2500 g) in the weeks following the time change. However, the data obtained allow us to affirm that the transition from solar time to summer time and vice versa does not have a statistically significant effect on the increase in the incidence of newborns born with a low birth weight (<2500 g) (*p* = 0.97). It is possible to extend the same statement to the other investigated weight classes (2500–4000 g: *p*-value = 0.45; >4000 g: *p* = 0.98). Thus, we can conclude that DST shift does not have a statistically significant impact on newborn birth weight.

The rationale for the analysis of the use of epidural analgesia in periods of exposure and control was to assess whether the desynchronization induced by DST that shifts the transition could play a role in the perception of pain. In this case also, the results obtained do not allow us to exclude that the differences found are attributable to chance (*p* = 0.64).

We took into consideration age and gestational age of this low risk population and we found that the mean age of women who delivered during the two weeks before DST was older than those who delivered during the two weeks post DST and that women’s age was independently associated with delivery during the two weeks post DST. We do not think that these findings could be of any relevant clinical significance due to the very small difference in age between the two groups. Moreover, these results could be related to the selection criteria aiming at evaluating only very low risk pregnancies.

We are aware of several limitations: (i) retrospective observational study; (ii) lack of perinatal complications—however, we selected only very low risk pregnancies; (iii) three years represent a short period of observation; (iv) unfortunately, we did not apply the methods used by László et al. [19] due to the different statistical package used for analysis—in fact, we used SPSS instead of SAS. Finally, (v) possible differences by race and ethnicity, since 35% of patients were not Italian. However, according to the last report of the Ministry for Health (year 2017) [26], in Italy, 21% of the births were related to mothers of non-Italian citizenship. This phenomenon is more widespread in the areas of the country with a greater foreign presence, i.e., in the Center–North, where more than 25% of births are from non-Italian mothers; in particular, in Emilia-Romagna and Lombardy, approximately 31% of births refer to foreign mothers. The most represented geographical areas of origin are Africa (27.7%), European Union (24.4%), Asia (18.1%), and South America (7.5%) [26].

However, the present study has also several strengths: (i) the large sample size, (ii) the quality of the data, and (iii) the rigorous methodology. The sample analyzed is representative of women characterized by a very low risk pregnancy, and the Emilia-Romagna region is characterized by one the best regional health systems in Italy. The choice of performing the study in a specific Italian region allowed us to obtain a reliable, good, complete, uniform, and coded source of data. The Childbirth Assistance Certificate is unique for the entire regional territory, and its compilation is mandatory following each birth in the region, independent of the structure in which it takes place.

## 5. Conclusions

This study shows no differences in the number of deliveries in the weeks following the change of time compared to those that precede it, neither during the Spring nor in the Autumn. This finding provides further confirmation to previous data obtained at quite different conditions of latitude, climate, and light exposure. It is possible that the multihormonal etiology of labor may explain this phenomenon. It would be interesting to extend the study to different latitudes and ethnicities, in order to verify whether our findings could be generalized or not.

## Figures and Tables

**Table 1:** The table shows the exclusion criteria applied on the sample.

Exclusion Criteria
Not low risk pregnancy
Not cephalic presentation
Induction of labor
Multiple pregnancy
Fetal growth defects
Medically Assisted Procreation (MAP) pregnancies
Fetal anomalies
Stillbirth
Elective Cesarean section

**Table 2:** Birth rates in the years 2016, 2017, and 2018 are shown.

Year of Study	Spring Shift		Autumn Shift	
	Control Period	Exposure Period	Control Period	Exposure Period
**2016**	18.39	17.42	20.89	21.21
**2017**	17.52	14.04	19.03	19.00
**2018**	16.27	17.99	17.03	17.12

Data are expressed as * 10 ^3^.

**Table 3:** Summarized data of results of primary outcome.

Primary Outcome	Spring Shift		Autumn Shift		CHI-SQUARE	*p*-Value (α = 5%)
	Control Period	Exposure Period	Control Period	Exposure Period		
**Number of deliveries**	1781	1727	1950	1957	0.546	0.46
**Number of spontaneous deliveries**	1588	1536	1744	1716	0.120	0.73

**Table 4:** Summarized data of results of secondary outcomes.

Secondary Outcome	Spring Shift		Autumn Shift		CHI-SQUARE	*p*-Value (α = 5%)
	Control Period	Exposure Period	Control Period	Exposure Period		
Delivery at term	1700	1652	1860	1849	0.227	0.63
Vaginal delivery at term	1516	1468	1663	1623	0.024	0.89
Post-term delivery	22	14	18	26	2.474	0.12
Preterm delivery	52	55	65	74	0.082	0.78
**Type of delivery**						
Cesarean Section	103	83	104	119	3.099	0.08
Operative delivery	90	108	102	122	0.0003	0.99
**Time of delivery**						
Morning (5:01 a.m. to 1:00 p.m.)	605	605	667	674	0.017	0.89
Afternoon (1:01 p.m. to 9:00 p.m.)	526	506	600	585	0.025	0.87
night (9:01 p.m. to 5:00 a.m.)	650	615	681	696	0.980	0.32
**Birth weight**						
Less than 2500 g	30	28	43	43	0.001	0.97
Between 2500 and 4000 g	1625	1579	1771	1785	0.564	0.45
More than 4000 g	126	120	136	129	0.0005	0.98
**5-min Apgar at birth**						
Apgar 7 at 5 min	11	6	10	12	0.760	0.38
Apgar 8 at 5 min	22	29	28	36	0.015	0.90
Apgar 9 at 5 min	242	245	270	254	0.339	0.56
Apgar 10 at 5 min	1502	1441	1632	1643	0.899	0.34
**Use of analgesia in labor**	313	299	355	357	0.217	0.64

**Table 5:** Logistic regression analysis considering the period including the two weeks pre and post DST as the dependent variable and the other parameters investigated the independent ones.

Variables	Odds Ratios	95% Confidence Intervals	*p*
Maternal age	1.010	1.002–1.019	0.021
Gestational age	1.005	0.970–1.041	0.801
Type of delivery	0.917	0.780–1.079	0.295
Time of delivery	1.044	0.930–1.172	0.466
Birth weight	1.000	1.000–1.000	0.639
5-min Apgar at birth	1.025	0.947–1.109	0.545
Use of analgesia in labor	1.023	0.900–1.163	0.726
